# Peroxisome Proliferator-Activated Receptor γ Expression Is Inversely Associated with Macroscopic Vascular Invasion in Human Hepatocellular Carcinoma

**DOI:** 10.3390/ijms17081226

**Published:** 2016-07-29

**Authors:** Hui-Tzu Hsu, Ming-Ta Sung, Chih-Chun Lee, Yin-Ju Kuo, Chin-Wen Chi, Hsin-Chen Lee, Cheng-Yuan Hsia

**Affiliations:** 1 Department and Institute of Pharmacology, School of Medicine, National Yang-Ming University, Taipei 112, Taiwan; sky7486m@gmail.com (H.-T.H.); philamo@gmail.com (C.-C.L.); chinwenchi@gmail.com (C.-W.C.); hclee2@ym.edu.tw (H.-C.L.); 2 Program in Molecular Medicine, National Yang-Ming University and Academia Sinica, Taipei 112, Taiwan; 3 Department of Medical Research, Taipei Veterans General Hospital, Taipei 112, Taiwan; sungmd@gmail.com; 4 Department of Surgery, Koo Foundation Sun Yat-Sen Cancer Center, Taipei 112, Taiwan; 5 Department of Pathology, Taipei Veterans General Hospital, Taipei 112, Taiwan; yjkuo2@vghtpe.gov.tw; 6 Faculty of Medicine, School of Medicine, National Yang-Ming University, Taipei 112, Taiwan; 7 Department of Surgery, Taipei Veterans General Hospital, Taipei 112, Taiwan

**Keywords:** PPARγ, macroscopic vascular invasion, hepatocellular carcinoma

## Abstract

Peroxisome proliferator-activated receptor γ (PPARγ) is a ligand-activated nuclear receptor that regulates cellular lipid and glucose metabolism and also plays an inhibitory role in various cancers. However, the role of PPARγ in hepatocellular carcinoma (HCC) remains controversial. This study aimed to investigate the prognostic value of PPARγ in HCC and its role in inhibiting tumor progression, namely, HCC cell growth, migration, and angiogenesis. Immunohistochemical PPARγ staining was examined in 83 HCC specimens to investigate the clinicopathological correlations between PPARγ expression and various parameters. The functional role of PPARγ was determined via PPARγ overexpression and knockdown in HCC cells. Patients with low HCC tissue PPARγ expression were significantly younger (*p* = 0.006), and exhibited more tumor numbers (*p* = 0.038), more macroscopic vascular invasion (MVI) (*p* = 0.008), and more advanced TNM (size of primary tumor, number of regional lymph nodes, and distant metastasis) stages at diagnosis (*p* = 0.013) than patients with high HCC tissue PPARγ expression. PPARγ knockdown increased HCC cell growth, migration, and angiogenesis, while PPARγ overexpression reduced HCC cell growth, migration, and angiogenesis. These results suggest that low PPARγ expression is an independent predictor of more MVI in HCC patients. PPARγ contributes to the suppression of HCC cell growth, migration, and angiogenesis. Therefore, PPARγ may be a therapeutic target in HCC patients.

## 1. Introduction

Hepatocellular carcinoma (HCC) is a major cause of cancer-related death worldwide [1], particularly in Asia and Africa. Hepatitis B virus (HBV) and hepatitis C virus (HCV) infection are two risk factors for HCC development [2]. Surgical resection is curative in HCC, but post-treatment recurrence and distant metastasis remain the major causes of death affected patients [3]. Therefore, understanding the molecular mechanism underlying HCC invasiveness is important for developing prognostic markers and new therapeutic targets for preventing tumor recurrence and improving survival rates.

Peroxisome proliferator-activated receptor γ (PPARγ) is a ligand-activated nuclear hormone receptor that regulates insulin sensitivity, glucose metabolism, and inflammation in liver tissue, adipose tissue, and skeletal muscle tissue. Ligand binding to PPARγ triggers PPARγ and retinoid X receptor (RXR) heterodimerization, which may recruit co-activators or co-repressors to PPAR response elements (PPREs) within the promoters of PPARγ target genes and regulate their transcription [4]. PPARγ activity can be induced by natural and synthetic ligands. 15-Deoxy-∆^12,14^-prostaglandin J_2_ (15d-PGJ_2_) is a natural PPARγ ligand [5] and thiazolidinediones (TZDs), such as rosiglitazone, troglitazone, and pioglitazone are synthetic PPARγ ligands [6]. Accumulating evidence indicates that PPARγ plays a critical role in cancer cell growth [7], migration [8], invasion [9], and apoptosis [10]. PPARγ combined with its ligands to exert inhibitory effects on HCC cell growth, migration, and metastasis in vitro and in mouse models [11,12,13]. In addition, Krüppel-like factor 4 (KLF4), a tumor suppressor in HCC [14,15,16], has been reported to be up-regulated by the PPARγ agonist troglitazone and promotes cell cycle arrest in colorectal cancer cells [17]. These findings indicate that PPARγ collaborates with KLF4 to regulate HCC tumorigenesis and cancer progression.

To date, only a few studies have described the changes in PPARγ expression in human HCC tissues. Schaefer et al. reported that high PPARγ protein expression was detected in 20 HCC tissues, but no expression was detected in non-tumorous livers [18]. Similarly, another group reported that the *PPAR*γ mRNA expression was significantly increased in 16 HCC tissues compared with the non-tumorous livers [19]. However, a third group reported that PPARγ protein expression was decreased in HCC tissues compared with non-tumorous livers in 20 HCC patients [20]. These results suggest that not only are the findings regarding PPARγ expression in HCC controversial, but the clinicopathological significance of PPARγ in human HCC also still unclear. The relationship between PPARγ expression and patient survival after curative treatment also remains unclear.

In this study, we examined the relationship between PPARγ protein expression and various clinicopathological variables such as age, tumor number, macroscopic vascular invasion (MVI), TNM (size of primary tumor, number of regional lymph nodes, and distant metastasis) stage, and survival rate in 83 HCC patients, who have underwent surgical resection. We also investigated the role of PPARγ in cell proliferation, migration, and angiogenesis via its overexpression and knockdown of PPARγ (peroxisome proliferator-activated receptor γ) in HCC cells.

## 2. Results

### 2.1. Peroxisome Proliferator-Activated Receptor γ (PPARγ) Protein Expression in Human Hepatocellular Carcinoma (HCC) Tissues and Associated Clinicopathological Characteristics

**Figure 1:**
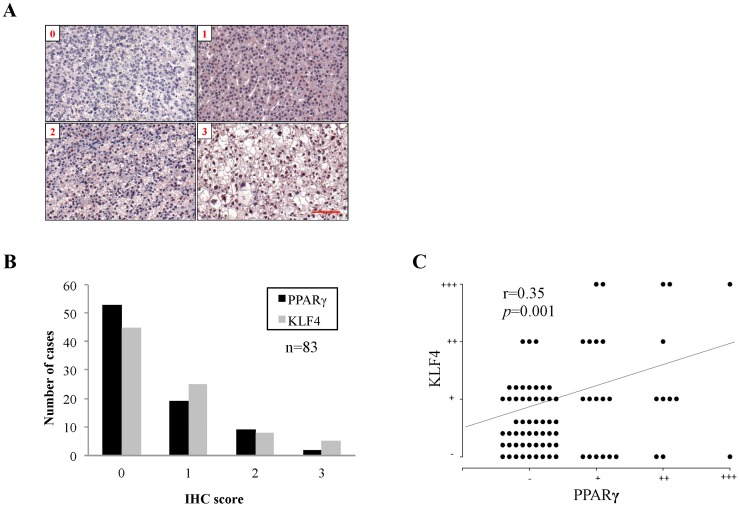
Peroxisome proliferator-activated receptor γ (PPARγ) and Krüppel-like factor 4 (KLF4) protein expression in human hepatocellular carcinoma (HCC) tissues: (**A**) representative views indicated PPARγ expression scores ranging from 0 to 3, as determined by immunohistochemistry (IHC); (**B**) the case number bar chart; and (**C**) correlation plot were generated using PPARγ and KLF4 staining scores from 83 human HCC tissue samples. Scale bar represents 100 μm.

To determine the clinicopathological significance of PPARγ protein expression in HCC, we examined PPARγ and downstream KLF4 expression in 83 HCC tissue samples via immunohistochemistry (IHC). IHC results were scored from 0 to 3 to indicate the percentages of cells with positive PPARγ staining (Figure 1A). Of the 83 HCC tissue samples, 30 (36.1%) exhibited positive PPARγ staining (score > 0), and were considered to have high PPARγ expression, and 53 (63.9%) exhibited negative PPARγ staining (score = 0), and were considered to have low PPARγ expression (Figure 1B). A similar trend was observed regarding KLF4 expression (Figure 1B). Moreover, IHC staining of KLF4 and PPARγ revealed a significantly positive correlation between the expression levels of the two proteins (*r* = 0.35, *p* = 0.01, Chi-square < 0.001) (Figure 1C). Various clinicopathological parameters, including patient age (*p* = 0.006), tumor number (*p* = 0.038), MVI (*p* = 0.008), and TNM stage (*p* = 0.013), exhibited significant associations with PPARγ expression (Table 1). Notably, MVI was independently associated with PPARγ expression based on the results of the multiple logistic regression analysis, indicating that low PPARγ expression is independently predictive of more MVI in HCC patients. In this study, patients with MVI exhibited significantly worse survival than patients without MVI (Figure S2). Subgroup analysis showed that patients with high PPARγ expression and no MVI exhibited superior disease-free survival (DFS) and overall survival (OS) rates (40.7% and 46.1%, respectively) than patients with low PPARγ expression and MVI (23.5% and 39.2%, respectively), although this difference was not statistically significant (*p* = 0.202 and *p* = 0.720, respectively) (Figure S3). These results suggest that low PPARγ expression is significantly correlated with poor clinicopathological findings in HCC patients.

### 2.2. PPARγ Suppresses HCC Cell Proliferation 

**Figure 2:**
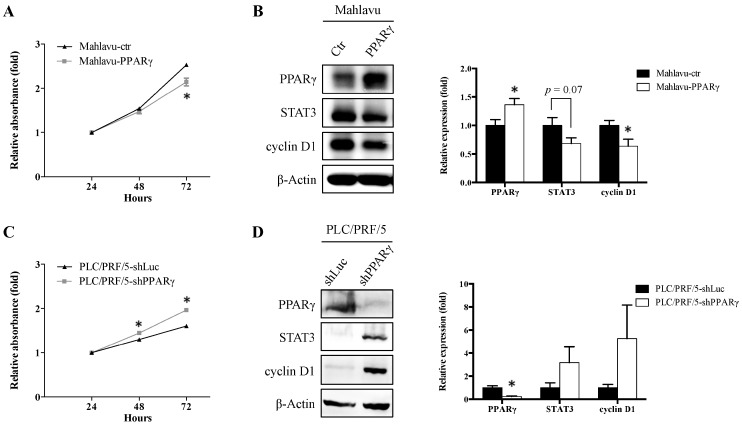
Effects of PPARγ overexpression and knockdown on cell proliferation and PPARγ downstream target protein expression in Mahlavu and PLC/PRF/5 HCC cells, respectively. (**A**,**C**) The cell proliferation rates of Mahlavu-ctr, Mahlavu-PPARγ, PLC/PRF/5-shLuc, and PLC/PRF/5-shPPARγ cells were analyzed by SRB assay; (**B**,**D**) the expression of PPARγ downstream target proteins STAT3 and cyclin D1 was analyzed by Western blot and the quantification results are shown. * *p* < 0.05 indicates a significant difference from vector control cells at the same time point.

Given that PPARγ is significantly associated with important HCC diagnostic and clinicopathological variables, we characterized its function in HCC via in vitro assays. Endogenous PPARγ and E-cadherin expression levels were examined in various HCC cell lines, including PLC/PRF/5, SK-Hep1, and Mahlavu cells (Figure S1). Our results revealed that Mahlavu cells, which are poorly differentiated and highly migratory, exhibited both low PPARγ and E-cadherin expression, whereas PLC/PRF/5 cells, which are well-differentiated and less migratory, exhibited both high PPARγ and high E-cadherin expression. To simulate different clinical scenarios, we overexpressed PPARγ in Mahlavu cells via a retrovirus-mediated gene transfer. We also knock down PPARγ expression in PLC/PRF/5 cells via a lentivirus-mediated gene transfer. Moreover, STAT3 and cyclin D1 protein expression was analyzed because both proteins are downstream targets of PPARγ-mediated signaling and are essential for cell cycle progression [21,22]. We found that PPARγ-overexpressing cells (Mahlavu-PPARγ) exhibited decreased cell growth rates (Figure 2A) and reduced STAT3 and cyclin D1 expression compared with vector control cells (Mahlavu-ctr) (Figure 2B). In contrast, PPARγ knockdown cells (PLC/PRF/5-shPPARγ) exhibited increased cell growth rates (Figure 2C) and higher STAT3 and cyclin D1 expression compared with luciferase control cells (PLC/PRF/5-ctr) (Figure 2D). Taken together, these findings indicate that PPARγ suppresses HCC cell proliferation, and down-regulates STAT3 and cyclin D1 expression.

### 2.3. PPARγ Inhibits HCC Cell Migration

**Figure 3:**
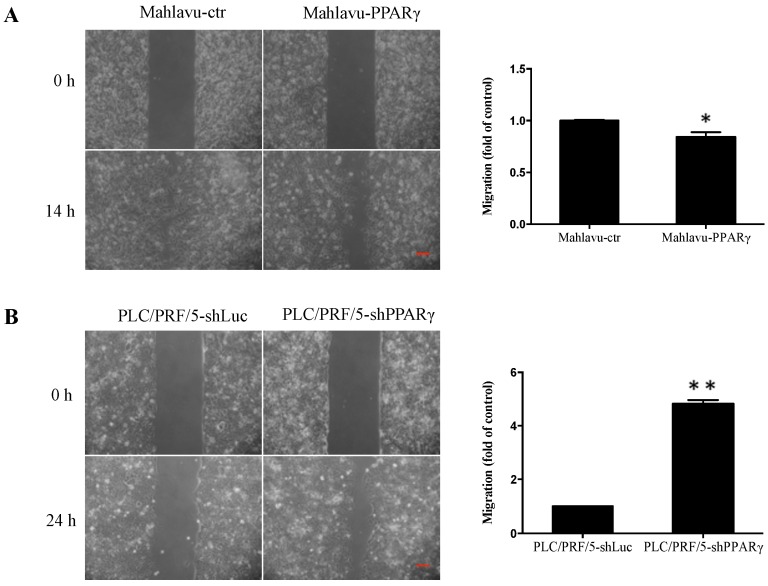
Effects of PPARγ overexpression and knockdown on cell migration of Mahlavu cells and PLC/PRF/5 cells, respectively. (**A**) Cell migration abilities of Mahlavu-ctr and Mahlavu-PPARγ cells were analyzed over 14 h by wound healing assay; (**B**) cell migration abilities of PLC/PRF/5-shLuc and PLC/PRF/5-shPPARγ cells were assessed over 24 h by wound healing assay. Relative quantification data are expressed as the mean ± SEM (standard error of the mean) from three independent experiments. * *p* < 0.05 and ** *p* < 0.001 indicate significant differences compared with vector control cells. Scale bar represents 100 μm.

We investigated the effect of PPARγ on HCC cell migration and found that PPARγ-overexpressing cells (Mahlavu-PPARγ) exhibited a 16% decrease in cell migration compared with control cells as determined via wound healing assay (Figure 3A). Conversely, PPARγ-knockdown cells (PLC/PRF/5-shPPARγ) exhibited significant four-fold increases in migration compared with control cells (Figure 3B). These results suggest that PPARγ inhibits HCC cell migration.

### 2.4. PPARγ Decreases HCC Cell Angiogenesis

**Figure 4:**
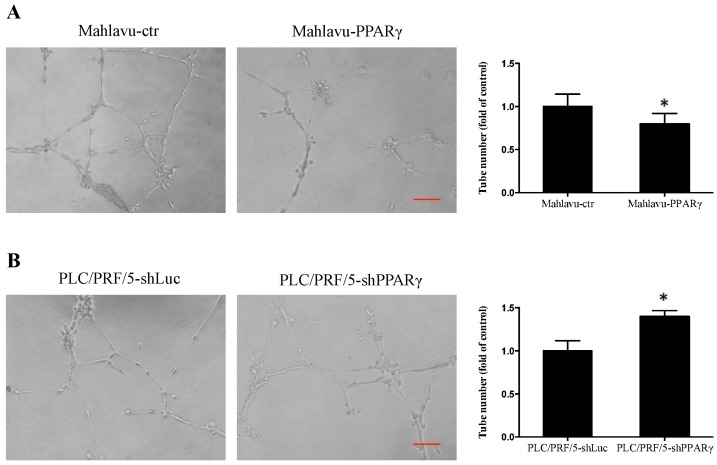
Effects of PPARγ overexpression and knockdown on in vitro human umbilical vein endothelial cells (HUVEC) tube formation in Mahlavu and PLC/PRF/5 cells, respectively. Conditioned medium was harvested from: (**A**) Mahlavu-ctr and Mahlavu-PPARγ; and (**B**) PLC/PRF/5-shLuc and PLC/PRF/5-shPPARγ cell cultures. The conditioned medium was used for HUVEC cell tube formation, and photos were taken after 6 h of incubation. Quantification data are expressed as the mean ± SEM from three independent experiments. * *p* < 0.05 indicates significant differences compared with vector control cells. Scale bar represents 100 μm.

Table 1 shows that PPARγ expression was inversely associated with MVI in human HCC patients and that MVI is associated with angiogenesis. We examined the effects of PPARγ on HCC cell angiogenesis. The results showed that PPARγ-overexpressing cell (Mahlavu-PPARγ)-conditioned medium decreased the number of vessel joints in human umbilical vein endothelial cells (HUVECs) by 20% compared with the control cell-conditioned medium (Figure 4A). In contrast, the PPARγ-knockdown cell (PLC/PRF/5-shPPARγ)-conditioned medium increased the number of vessel joints by 40% compared with the control cell-conditioned medium (Figure 4B). In addition, the PPARγ knockdown cells (PLC/PRF/5-shPPARγ) exhibited increased vascular endothelial growth factor (VEGF) expression compared with control cells; however, no significant differences in VEGF expression were observed between PPARγ-overexpressing cells (Mahlavu-PPARγ) and control cells (Figure S4). These results suggest that PPARγ inhibits HCC cell angiogenesis.

## 3. Discussions

This study was the first to analyze the clinicopathological significance of PPARγ in HCC using a relatively large sample size. We demonstrated that HCC patients with low PPARγ expression were significantly younger than 65 years old and exhibited more tumor numbers, more MVI, and more advanced TNM stages at diagnosis than HCC patients with high PPARγ expression. All of these clinicopathological factors are considered prognostic factors for patient survival. In particular, MVI has long been considered a major determining factor of TNM stage and patient survival and HCC patients exhibiting low PPARγ expression were independently predicted to have more MVI. In addition, we demonstrated that PPARγ inhibits HCC cell proliferation, migration, and angiogenesis. These results suggest that PPARγ functions as a tumor suppressor in HCC cells and may be a therapeutic target in HCC.

We found that 53 of 83 HCC samples exhibited negative PPARγ staining (score = 0) and that only 30 samples exhibited positive PPARγ staining (score > 0), indicating that most HCC tissues exhibited low or no PPARγ protein expression (Figure 1B). Consistent with the results from the Human Protein Atlas (www.proteinatlas.org), nine of 11 HCC tissue samples exhibited non-detectable PPARγ staining. However, previous studies demonstrated that PPARγ protein and gene expression varies in HCC tissues compared with normal tissues across different assays [18,19,20]. A study of colorectal cancer cells demonstrated that PPARγ regulates *KLF4* transcription by directly binding to the *KLF4* promoter [17]. Reduced KLF4 expression was also observed in HCC [14]. Our analysis revealed that a significantly positive correlation exists between PPARγ and KLF4 expression in HCC tissues, suggesting that PPARγ collaborates with KLF4 to facilitate tumor suppression.

HCC patients with MVI involving portal or hepatic veins exhibited an increased risk of tumor recurrence and worse prognoses after liver resection or transplantation than patients without extensive MVI [23]. MVI has also been suggested to be an independent predictor of recurrence after liver transplantation [24]. Consistent with these findings, our results also showed that HCC patients with MVI exhibited a significantly decreased in DFS rates compared with patients without MVI (Figure S2). HCC patients with high PPARγ expression and no MVI exhibited better DFS and OS than patients with low PPARγ expression and MVI (Figure S3). These results indicated that PPARγ expression is inversely associated with tumor development, namely, the number of tumor foci, the extent of MVI, and the TNM stage. Our findings indicate that low PPARγ expression is significantly associated with a poor prognosis in HCC patients.

In this study, PPARγ expression was negatively associated with TNM stage and MVI in HCC tissues. To simulate this clinical scenario and determine the functional role of PPARγ in HCC, we used the poorly differentiated and relatively low PPARγ-expressing HCC cell line Mahlavu to overexpress PPARγ. We also used the well-differentiated and relatively high PPARγ-expressing HCC cell line PLC/PRF/5 to knock down PPARγ. We noted decreased growth and decreased STAT3 and cyclin D1 expression in PPARγ-overexpressing HCC cells (Figure 2A). Conversely, we noted increased growth and increased STAT3 and cyclin D1 expression in PPARγ knockdown HCC cells (Figure 2B). These results are consistent with those of previous studies involving PPARγ-deficient (PPARγ^+/−^) mice and different cell lines. Yu et al. demonstrated that PPARγ-deficient (PPARγ^+/−^) mice were susceptible to diethylnithrosamine-induced liver carcinogenesis compared with wild-type mice (PPARγ^+/+^), suggesting that PPARγ functions as a tumor suppressor in hepatocarcinogenesis [12]. In pancreatic cancer cells, PPARγ activation by PPARγ agonists suppressed *STAT3* expression through transcriptional repression to inhibit cell growth [21]. In addition, PPARγ activation by a synthetic PPARγ agonist, pioglitazone, resulted in growth inhibition and decreased cyclin D1 expression in human HCC SMMC-7721 and HepG2 cells [22]. Together, these data suggest that PPARγ inhibits cell growth by down-regulating STAT3 and cyclin D1 expression in HCC cells.

Moreover, we observed PPARγ inhibited HCC cell migration (Figure 3) and in vitro angiogenesis (Figure 4). Previous studies involving human HCC MHCC97L and BEL-7404 cells suggested that PPARγ overexpression suppressed cell migration and invasion by down-regulating matrix metalloproteinase (MMP) 9, MMP13, and heparanase (HPSE) expression, while up-regulating E-cadherin and tissue inhibitor of metalloproteinase (TIMP) 3 expression [11]. PPARγ overexpression decreased cell invasion by up-regulating plasminogen activator inhibitor-1 (PAI-1) expression in HepG2 cells [13]. Moreover, PPARγ ligands facilitated cell cycle arrest, apoptosis, and metastasis inhibition in HCC via multiple pathways [25]. In addition, PPARγ ligands have also been shown to exert anti-angiogenic effects and to decrease VEGF expression in different types of cancers, such as glioblastoma and Lewis lung carcinoma cells [26,27]. It has been reported that the human VEGF promoter contains a PPRE. Treatments with PPARγ ligands, such as rosiglitazone and 15-Deoxy-∆^12,14^-prostaglandin J_2_, repressed *VEGF* gene expression through direct binding to the *VEGF* PPRE promoter in human endometrial cells [28]. These results suggest that the absence of PPARγ and the loss of the repressor in the VEGF promoter may result in increased VEGF expression, indicating that PPARγ plays an inhibitory role in the HCC cell growth, migration, and angiogenesis; thus, PPARγ may be a therapeutic target for HCC treatment.

In conclusion, we demonstrated for the first time that low PPARγ expression was significant associated with patient age, tumor number, MVI, and TNM stage in HCC patients. In vitro experiments showed that PPARγ suppresses tumor progression, namely, growth, migration, and angiogenesis in HCC cells. Cyclin D1 and STAT3 may be involved in PPARγ-mediated signaling pathways that inhibit HCC cell growth. Absence of PPARγ expression may lead to increased invasiveness in HCC. Our findings indicate that PPARγ expression may determine patient prognosis in HCC and that PPARγ may serve as a therapeutic target for HCC treatment.

## 4. Materials and Methods

### 4.1. Human Tissue Specimens and Patient Information

Eighty-three patients who underwent curative liver resection for HCC in Taipei Veteran General Hospital were enrolled in the study. These patients ranged from 28 to 88 years (average of 61 ± 14 years). Sixty patients were male and 23 were female. A total of 83 paraffin-embedded HCC samples were obtained from the surgical tissue bank of Taipei Veterans General Hospital, Taiwan. Institutional review board (IRB) approval was obtained for this retrospective study (IRB No: 2013-02-031BC). HCC was morphologically classified, according to the World Health Organization guideline. Representative paraffin blocks were obtained from pathologically confirmed waxed-preserved HCC specimens. The paraffin blocks were then used to generate a tissue array and cut into 3-μm-thick sections for further investigation.

### 4.2. Immunohistochemical

PPARγ and KLF4 protein expression was examined by IHC using a DAKO LSAB2 Kit (Agilent Technologies, Produktionsvej, Denmark). Tissue sections were microwaved in sodium citrate buffer (10 mM, pH 6), treated with 3.0% H_2_O_2_ for 10 min and soaked with blocking solution for 10 min. The tissue sections were incubated overnight with antibodies specific for PPARγ (Santa Cruz Biotechnology, Dallas, TX, USA) and KLF4 (Atlas, Stockholm, Sweden) at dilutions of 1:100 at room temperature in a moist chamber. Then, the tissue section slides were washed in PBS and incubated with a biotin-labeled secondary antibody for 10 min, before being incubated with a streptavidin horseradish peroxidase (HRP)-conjugated secondary antibody for 10 min. After the sections were incubated with a 3,3-diaminobenzidine tetrahydrochloride (DAB) substrate chromogen for 10 min, the Mayer’s hematoxylin counterstain was applied for 10 min (Muto Pure Chemicals, Tokyo, Japan). Finally, mounting solution (Kaiser’s glycerol gelatin, Merck, Kenilworth, NJ, USA) was added to the sections, which were covered with cover slides for histological examination. A pathologist blinded patient clinicopathological data perform examination. The staining results were graded on a scale of 0 to 3 and represented as percentages of positively stained cells. A score of 0 indicated negative staining (<10%), a score of 1 indicated weak staining (10% to 25% of cells stained positive), a score of 2 indicated moderate staining (25% to 50% of cells stained positive), and a score of 3 indicated strong staining (greater than 50% of cells stained positive). A score of 0 indicated low PPARγ expression, and scores ranging from 1 to 3 were indicated high PPARγ expression.

### 4.3. Cell Culture

PLC/PRF/5, Mahlavu, and SK-Hep1 HCC cell lines (obtained from Cell Bank of Taipei Veterans General Hospital, Taipei, Taiwan) and HEK293T cells (ATCC, Manassas, VA, USA) were maintained in Dulbecco’s modified Eagle’s medium (DMEM) supplemented with 10% fetal bovine serum, 0.1 mM non-essential amino acids, 2 mM l-glutamine, and 1% penicillin/streptomycin in a humidified atmosphere containing with 5% CO_2_ at 37 °C. All cell culture reagents were obtained from Invitrogen (Carlsbad, CA, USA).

### 4.4. PPARγ Overexpression and Knockdown

Retroviral expression vectors carrying PPARγ full-length cDNA or pBABE-puro-PPARγ were constructed. HEK293T cells were co-transfected with these retroviral expression vectors and packaging plasmids (pAmpho and pCMV-VSV-G) using TurboFECT (Thermo Scientific, Waltham, MA, USA). Supernatants were collected 72 h after transfection and filtered. Mahlavu cells were infected with the retroiviral expression vectors in the presence of 8 μg/mL polybrene (Sigma-Aldrich, St. Louis, MO, USA). The lentiviral vector pLVO.1-shPPARG, which was used for PPARγ knockdown, was purchased from the RNAi core of Academia Sinica (Taipei, Taiwan). The oligonucleotide targeting human PPARγ was 5′-GACAACAGACAAATCACCATT-3′. The lentiviruses were generated by co-transfecting HEK293T cells with the indicated lentiviral expression vectors (pLVO.1-puro and pLVO.1-shPPARG) and packaging plasmids (pCMVΔR8.91 and pCMV-VSV-G) using TurboFECT (Thermo Scientific). Supernatants containing the lentiviruses were collected 72 h after transfection and filtered. PLC/PRF/5 cells were infected with lentiviruses in the presence of 8 μg/mL polybrene (Sigma-Aldrich). Stable clones of PPARγ-overexpressing and PPARγ-knockdown HCC cells were selected by puromycin (Sigma-Aldrich). The packaging plasmids pAmpho, pCMV-VSV-G, and pCMVΔR8.91 were purchased from the RNAi core of Academia Sinica.

### 4.5. Cell Proliferation Measurement

Sulforhodamine B (SRB) colorimetric analysis was used to measure cell proliferation. Cells were seeded at a density of 4 × 10^3^ cells/well in 96-well plates. First, the cells were fixed in cold 10% trichloroacetic acid (Sigma-Aldrich) at 4 °C for 1 h. After being washed with water and air-dried the fixed cells were incubated with 1% SRB (Sigma-Aldrich), dissolved in 1% acetic acid for 30 min. Unincorporated dye was removed by five rinses with 1% acetic acid. The protein-bound dye was extracted with 10 mM Tris and then optical absorbance was measured at a wavelength of 510 nm was measured by a spectrophotometer.

### 4.6. Western Blot Analyses 

Total proteins were extracted from cells using RIPA buffer (150 mM NaCl, 50 mM Tris-HCl, 0.25% sodium deoxycholate, 1% Triton X-100, 0.1% SDS), supplemented with a protease inhibitor cocktail (Calbiochem, San Diego, CA, USA). Proteins were separated via 10% SDS-PAGE gel and electrotransferred onto a polyvinylidene difluoride (PVDF) membrane. The PVDF membrane was blocked in 5% skimmed milk at room temperature for 1 h and probed with primary antibodies. PPARγ (Cell Signaling, Danvers, MA, USA), E-cadherin (Cell Signaling), signal transducer and activator transcription 3 (STAT3) (Cell Signaling), cyclin D1 (Millipore, Darmstadt, Germany), and β-actin (Sigma-Aldrich) antibodies were used to probe the proteins on the membrane at 4 °C overnight. After incubation with an HRP-conjugated secondary antibody (Jackson ImmunoResearch Laboratories, West Grove, PA, USA), the probed proteins were detected by an enhanced chemiluminescence system (Thermo Scientific), according to the manufacturer’s instructions.

### 4.7. Wound Healing Assay 

Changes in cell migration ability were assessed via in vitro wound healing assay with a Culture-Insert (Ibidi, Am Klopferspitz, Germany), which is a special sticky and biocompatible surface, the bottom side of which works like glue and avoids leaking. Cells were seeded into each well of the Culture-Insert and then incubated overnight at 37 °C and 5% CO_2_. After the cells attached and achieved confluence, the Culture-Insert was gently removed to allow cell migration. We measured the migratory areas of Mahlavu cells and PLC/PRF/5 cells for 14 and 24 h, respectively, after removing the Culture-Insert. Four bright field images were obtained at 100× magnification at the indicated time points, and Image J software (National Institute of Health, Bethesda, MD, USA) was used to analyze the migratory areas.

### 4.8. Matrigel Tube Formation Assay

HUVECs (human umbilical vein endothelial cells) were cultured in Medium 200 with Low Serum Growth Supplement (Gibco, Carlsbad, CA, USA) in a humidified atmosphere, with 5% CO_2_ at 37 °C. Briefly, 96-well culture plates were coated with 50 µL of Matrigel (BD Biosciences, San Jose, CA, USA) per well and the Matrigel was allowed to polymerize for 30 min. HCC cell-conditioned medium was harvested over 2 days, and HUVECs were separately suspended in Mahlavu-ctr, Mahlavu-PPARγ, PLC/PRF/5-shLuc, and PLC/PRF/5-shPPARγ cell-conditioned medium. HUVEC culture medium was subsequently added to the suspension at a 1:1 ratio. The HUVECs were then seeded onto the polymerized Matrigel-coated wells at a density of 10^4^ cells/100 μL per well. Bright field images were obtained after the cells were incubated for 6 h in a 37 °C incubator. Tube-like structures were detected under an inverted light microscope at 200× to evaluate in vitro angiogenesis. The numbers of vessel joints in five fields were counted.

### 4.9. Statistical Analyses 

A Chi-square test was used for categorical variables. Continuous variables were expressed as the mean ± SEM (standard error of the mean), and the differences between groups were evaluated by Student’s *t*-test. Multivariate analysis was performed using the logistic regression model. Correlations between variables were calculated by Spearman’s co-efficient method. Survival was calculated using the Kaplan–Meier method, and the survival differences were assessed by the log-rank test. SPSS software version 19 (IBM Corporation, Armonk, NY, USA) was used to perform the statistical analyses. Differences were considered statistically significant at *p* < 0.05.

## Figures and Tables

**Table 1:** Multivariate analysis of PPARγ expression in relation to clinicopathological findings in HCC patients.

Characteristic	PPARγ Expression (Percentage)		*p* Value
	Low (=0) *n* = 53	High (>0) *n* = 30	
Age			
≤65	39	13	0.006 *
>65	14	17	
Sex			
Male	39	21	0.726
Female	14	9	
Tumor size			
<3 cm	8	9	0.106
>3 cm	45	21	
HBsAg ^∆^			
(−)	15	14	0.053
(+)	38	14	
Anti-HCV ^∆^			
(−)	44	22	0.148
(+)	7	8	
Cell differentiation			
Well differentiated	3	3	0.63
Moderately differentiated	32	19	
Poorly differentiated	18	8	
Tumor number ^∆^			
Single	33	25	0.038 *
Multiple	18	4	
Liver cirrhosis			
No	42	21	0.344
Yes	11	9	
Chronic hepatitis ^∆^			
No	6	7	0.227
Yes	41	23	
Fibrosis ^∆^			
No	29	17	0.293
Yes	12	12	
MVI			
No	36	28	0.008 *
Yes	17	2	
Bile duct invasion			
No	49	29	0.649
Yes	4	1	
AFP ^∆^			
<20	19	16	0.139
>20	33	14	
TNM stage			
I + II	30	25	0.013 *
III	23	5	
DFS			
Event/all	34/53	18/30	0.967
5-year survival	34.6%	38%	
OS			
Event/all	25/53	15/30	0.349
5-year survival	55.3%	46.6%	

PPARγ: Peroxisome proliferator-activated receptor γ; HCC: hepatocellular carcinoma; HBsAg: hepatitis B virus surface antigen, Anti-HCV: anti-hepatitis C virus, MVI: macroscopic vascular invasion, AFP: α-fetoprotein, DFS: disease-free survival, OS: overall survival. * *p* < 0.05. ^∆^ indicates a missing number. TNM: size of primary tumor, number of regional lymph nodes, and distant metastasis.

## Supplementary Materials

Supplementary materials can be found at http://www.mdpi.com/1422-0067/17/8/1226/s1.

## References

[1] Torre L.A., Bray F., Siegel R.L., Ferlay J., Lortet-Tieulent J., Jemal A. (2015). Global cancer statistics, 2012. CA Cancer J. Clin..

[2] El-Serag H.B. (2012). Epidemiology of viral hepatitis and hepatocellular carcinoma. Gastroenterology.

[3] Tang Z.Y. (2001). Hepatocellular carcinoma—Cause, treatment and metastasis. World J. Gastroenterol..

[4] Desvergne B., Wahli W. (1999). Peroxisome proliferator-activated receptors: Nuclear control of metabolism. Endocr. Rev..

[5] Forman B.M., Tontonoz P., Chen J., Brun R.P., Spiegelman B.M., Evans R.M. (1995). 15-Deoxy-∆^12,14^-prostaglandin J_2_ is a ligand for the adipocyte determination factor PPARγ. Cell.

[6] Murphy G.J., Holder J.C. (2000). PPAR-γ agonists: Therapeutic role in diabetes, inflammation and cancer. Trends Pharmacol. Sci..

[7] Cao L.Q., Shao Z.L., Liang H.H., Zhang D.W., Yang X.W., Jiang X.F., Xue P. (2015). Activation of peroxisome proliferator-activated receptor-γ (PPARγ) inhibits hepatoma cell growth via downregulation of SEPT2 expression. Cancer Lett..

[8] Lee H.J., Su Y., Yin P.H., Lee H.C., Chi C.W. (2009). PPARγ/PGC-1α pathway in E-cadherin expression and motility of HepG2 cells. Anticancer Res..

[9] Gu J.J., Zhang J.H., Chen H.J., Wang S.S. (2016). MicroRNA-130b promotes cell proliferation and invasion by inhibiting peroxisome proliferator-activated receptor-γ in human glioma cells. Int. J. Mol. Med..

[10] Ohta K., Endo T., Haraguchi K., Hershman J.M., Onaya T. (2001). Ligands for peroxisome proliferator-activated receptor γ inhibit growth and induce apoptosis of human papillary thyroid carcinoma cells. J. Clin. Endocrinol. Metab..

[11] Shen B., Chu E.S., Zhao G., Man K., Wu C.W., Cheng J.T., Li G., Nie Y., Lo C.M., Teoh N. (2012). PPARγ inhibits hepatocellular carcinoma metastases in vitro and in mice. Br. J. Cancer.

[12] Yu J., Shen B., Chu E.S., Teoh N., Cheung K.F., Wu C.W., Wang S., Lam C.N., Feng H., Zhao J. (2010). Inhibitory role of peroxisome proliferator-activated receptor γ in hepatocarcinogenesis in mice and in vitro. Hepatology.

[13] Pang X., Wei Y., Zhang Y., Zhang M., Lu Y., Shen P. (2013). Peroxisome proliferator-activated receptor-γ activation inhibits hepatocellular carcinoma cell invasion by upregulating plasminogen activator inhibitor-1. Cancer Sci..

[14] Li Q., Gao Y., Jia Z., Mishra L., Guo K., Li Z., Le X., Wei D., Huang S., Xie K. (2012). Dysregulated kruppel-like factor 4 and vitamin D receptor signaling contribute to progression of hepatocellular carcinoma. Gastroenterology.

[15] Sung M.T., Hsu H.T., Lee C.C., Lee H.C., Kuo Y.J., Hua K., Hsia C.Y., Chi C.W. (2015). Kruppel-like factor 4 modulates the migration and invasion of hepatoma cells by suppressing TIMP-1 and TIMP-2. Oncol. Rep..

[16] Lin Z.S., Chu H.C., Yen Y.C., Lewis B.C., Chen Y.W. (2012). Kruppel-like factor 4, a tumor suppressor in hepatocellular carcinoma cells reverts epithelial mesenchymal transition by suppressing slug expression. PLoS ONE.

[17] Li S., Zhou Q., He H., Zhao Y., Liu Z. (2013). Peroxisome proliferator-activated receptor γ agonists induce cell cycle arrest through transcriptional regulation of kruppel-like factor 4 (KLF4). J. Biol. Chem..

[18] Schaefer K.L., Wada K., Takahashi H., Matsuhashi N., Ohnishi S., Wolfe M.M., Turner J.R., Nakajima A., Borkan S.C., Saubermann L.J. (2005). Peroxisome proliferator-activated receptor γ inhibition prevents adhesion to the extracellular matrix and induces anoikis in hepatocellular carcinoma cells. Cancer Res..

[19] Lin Y.M., Velmurugan B.K., Yeh Y.L., Tu C.C., Ho T.J., Lai T.Y., Tsai C.H., Tsai F.J., Tsai C.H., Huang C.Y. (2013). Activation of estrogen receptors with E2 downregulates peroxisome proliferator-activated receptor γ in hepatocellular carcinoma. Oncol. Rep..

[20] Yu J., Qiao L., Zimmermann L., Ebert M.P., Zhang H., Lin W., Rocken C., Malfertheiner P., Farrell G.C. (2006). Troglitazone inhibits tumor growth in hepatocellular carcinoma in vitro andin vivo. Hepatology.

[21] Vitale G., Zappavigna S., Marra M., Dicitore A., Meschini S., Condello M., Arancia G., Castiglioni S., Maroni P., Bendinelli P. (2012). The PPAR-γ agonist troglitazone antagonizes survival pathways induced by stat-3 in recombinant interferon-β treated pancreatic cancer cells. Biotechnol. Adv..

[22] Yang Y., Zhao L.H., Huang B., Wang R.Y., Yuan S.X., Tao Q.F., Xu Y., Sun H.Y., Lin C., Zhou W.P. (2015). Pioglitazone, a PPARγ agonist, inhibits growth and invasion of human hepatocellular carcinoma via blockade of the rage signaling. Mol. Carcinog..

[23] Faber W., Stockmann M., Kruschke J.E., Denecke T., Bahra M., Seehofer D. (2014). Implication of microscopic and macroscopic vascular invasion for liver resection in patients with hepatocellular carcinoma. Dig. Surg..

[24] Roayaie S., Frischer J.S., Emre S.H., Fishbein T.M., Sheiner P.A., Sung M., Miller C.M., Schwartz M.E. (2002). Long-term results with multimodal adjuvant therapy and liver transplantation for the treatment of hepatocellular carcinomas larger than 5 centimeters. Ann. Surg..

[25] Wu C.W., Farrell G.C., Yu J. (2012). Functional role of peroxisome-proliferator-activated receptor γ in hepatocellular carcinoma. J. Gastroenterol. Hepatol..

[26] Panigrahy D., Huang S., Kieran M.W., Kaipainen A. (2005). PPARγ as a therapeutic target for tumor angiogenesis and metastasis. Cancer Biol. Ther..

[27] Panigrahy D., Singer S., Shen L.Q., Butterfield C.E., Freedman D.A., Chen E.J., Moses M.A., Kilroy S., Duensing S., Fletcher C. (2002). PPARγ ligands inhibit primary tumor growth and metastasis by inhibiting angiogenesis. J. Clin. Investig..

[28] Peeters L.L., Vigne J.L., Tee M.K., Zhao D., Waite L.L., Taylor R.N. (2005). PPAR γ represses VEGF expression in human endometrial cells: Implications for uterine angiogenesis. Angiogenesis.

