# Supplementary material for: Peroxisome Proliferator-Activated Receptor γ Expression Is Inversely Associated with Macroscopic Vascular Invasion in Human Hepatocellular Carcinoma
Source: Int J Mol Sci. 2016 Jul 29;17(8):1226. doi: 10.3390/ijms17081226 (PMC5000624; doi:10.3390/ijms17081226)
Supplement: Supplementary file 1 [file ijms-17-01226-s001.pdf]

Supplementary Materials: Peroxisome Proliferator-Activated Receptor γ Expression is Inversely Associated with Macroscopic Vascular Invasion in Human Hepatocellular Carcinoma

Hui-Tzu Hsu, Ming-Ta Sung, Chih-Chun Lee, Yin-Ju Kuo, Chin-Wen Chi, Hsin-Chen Lee and Cheng-Yuan Hsia

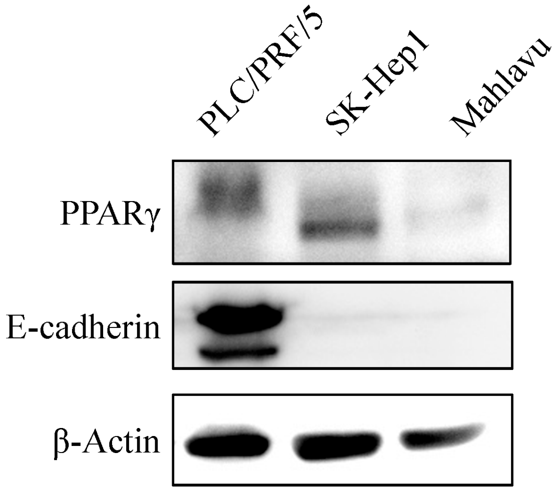

Figure S1. Endogenous peroxisome proliferator-activated receptor γ (PPARγ) and E-cadherin expression in different hepatocellular carcinoma (HCC) cell lines. Western blot was used to detect endogenous PPARγ and E-cadherin expression in PLC/PRF/5, SK-Hep1, and Mahlavu HCC cell lines.

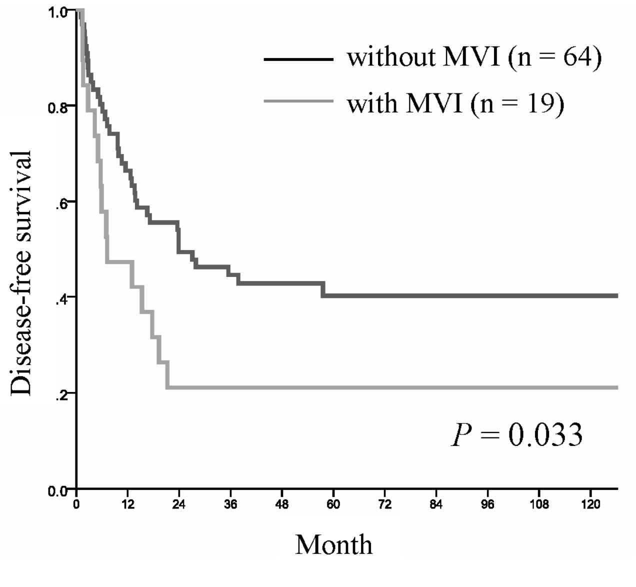

Figure S2. The 5-year disease-free survival (DFS) curves of patients underwent curative liver resection for HCC. Kaplan–Meier curves for DFS according to with (n = 19) or without (n = 64) macroscopic vascular invasion (MVI) in HCC patients.

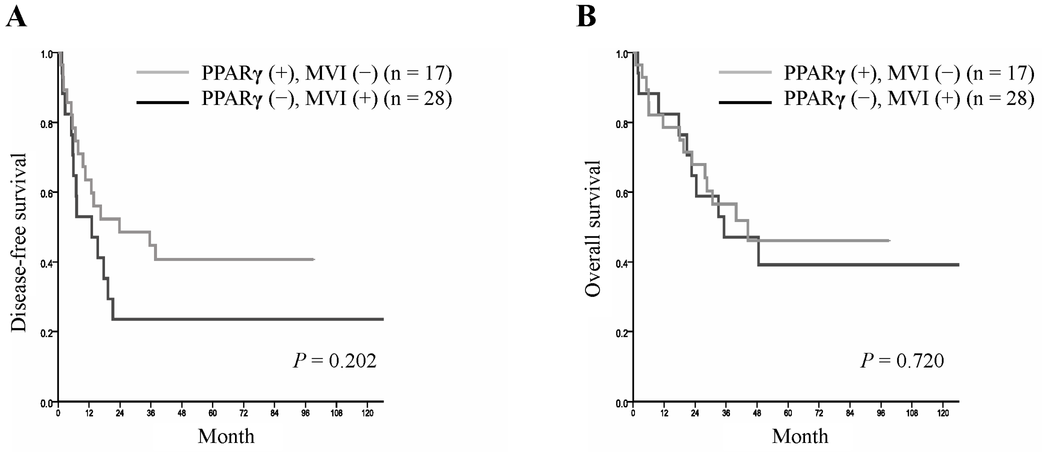

Figure S3. The 5-year disease-free survival (DFS) and overall survival (OS) analyses of HCC patients with or without MVI according to PPARγ expression. (**A**) Kaplan–Meier curves for DFS according to patients with high PPARγ expression and without macroscopic vascular invasion (MVI) (n = 17) and patients with low PPARγ expression and with MVI (n = 28); (**B**) Kaplan–Meier curves for OS according to patients with high PPARγ expression and without MVI (n = 17) and patients with low PPARγ expression and with MVI (n = 28).

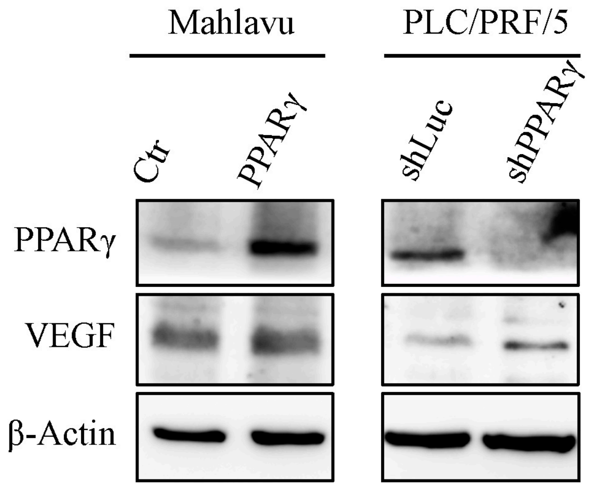

Figure S4. Vascular endothelial growth factor (VEGF) expression in PPARγ-overexpressed and PPARγ knockdown HCC cells. Western blot was used to examine VEGF expression in Mahlavu-ctr, Mahlauv-PPARγ, PLC/PRF/5-shLuc, and PLC/PRF/5-shPPARγ cells.
